# Design of a Tumorigenicity Test for Induced Pluripotent Stem Cell (iPSC)-Derived Cell Products

**DOI:** 10.3390/jcm4010159

**Published:** 2015-01-14

**Authors:** Shin Kawamata, Hoshimi Kanemura, Noriko Sakai, Masayo Takahashi, Masahiro J. Go

**Affiliations:** 1 Research and Development Center for Cell Therapy, Foundation for Biomedical Research and Innovation, TRI#308 1-5-4, Minatojima-Minamimachi, Chuo-ku, Kobe 650-0047, Japan; E-Mails: kanemura@fbri.org (H.K.); go@fbri.org (M.J.G.); 2 Laboratory for Retinal Regeneration, RIKEN Center for Developmental Biology, 2-2-3, Minatojima-Minamimachi, Chuo-ku, Kobe 650-0047, Japan; E-Mails: noriko-sakai@cdb.riken.jp (N.S.); mretina@cdb.riken.jp (M.T.)

**Keywords:** PSC-derived cell therapy, iPSC, RPE, tumorigenicity test

## Abstract

Human Pluripotent Stem Cell (PSC)-derived cell therapy holds enormous promise because of the cells’ “unlimited” proliferative capacity and the potential to differentiate into any type of cell. However, these features of PSC-derived cell products are associated with concerns regarding the generation of iatrogenic teratomas or tumors from residual immature or non-terminally differentiated cells in the final cell product. This concern has become a major hurdle to the introduction of this therapy into the clinic. Tumorigenicity testing is therefore a key preclinical safety test in PSC-derived cell therapy. Tumorigenicity testing becomes particularly important when autologous human induced Pluripotent Stem Cell (iPSC)-derived cell products with no immuno-barrier are considered for transplantation. There has been, however, no internationally recognized guideline for tumorigenicity testing of PSC-derived cell products for cell therapy. In this review, we outline the points to be considered in the design and execution of tumorigenicity tests, referring to the tests and laboratory work that we have conducted for an iPSC-derived retinal pigment epithelium (RPE) cell product prior to its clinical use.

## 1. Introduction

Several notable clinical trials using human Pluripotent Stem Cell (PSC)-derived cell products have been conducted recently. In the first, Geron used embryonic stem cell (ESC)-derived oligodendrocyte progenitor cells (GRNOPC1) for treatment of acute spinal cord injury [1]. Advanced Cell Technology initiated a study in which ESC-derived retinal pigment epithelium (RPE) was used for treatment of Stargardt’s disease and dry type Age-related Macular Degeneration (AMD) [2]. More recently, a clinical study for wet type AMD using induced Pluripotent Stem Cell (iPSC)-derived RPE was started at Riken CDB [3,4,5].

While clinical applications are moving forward, there are concerns that transplantation of differentiated PSC might lead to the formation of tumors in the recipient. Thus, examination of this possible outcome of transplantation is critically important. Cell transplantation or infusion therapy is distinctly different from drug administration. One must consider that transplanted or infused cells can survive for long periods in the host and may form tumors at the site of transplantation or at distal sites. The extent of tumor formation can be influenced by the microenvironment at the transplantation site or the ultimate homing site of the host. Furthermore, once a tumor has formed, it may influence the physical condition of the host through secreted factor(s) [6].

The aforementioned aspects of cell therapy must be addressed with animal transplantation studies prior to clinical use. Tumorigenicity tests that can assess the tumor-forming potential of transplanted cells are particularly important in the case of PSC-based cell therapies. As PSC have “unlimited” proliferation potential as undifferentiated stem cells, they can generate teratomas if they remain in the final product. The chance of generating a teratoma will increase if the procedure uses an autologous iPSC-derived cell product that presents no immunologic barrier. PSC might accumulate chromosomal abnormalities by selecting cells with unusual proliferative advantages over a long culture period. Lund *et al.* reported that some 13% of ESC and iPSC maintained in research labs worldwide demonstrated some type of genetic abnormality [7]. For that reason, the timely assessment of the genetic stability of PSC is of major interest for both research labs and clinical PSC banks. In addition, it is important to assess the potential for differentiation resistance due to incomplete reprogramming or a differentiation bias due to epigenetic memory when iPSC-based therapy is considered. In this context, it is necessary to assess the tumor-forming potential of non-terminally differentiated cells as well.

Information regarding genetic stability, gene expression, differentiation marker expression, cell growth rate and how cells were generated must be collected and evaluated prior to commencement of tumorigenicity testing. Next, it is necessary to have a clear idea about the scope and objective of related safety parameters: toxicology tests, Proof of Concept (POC) tests, biodistribution tests and tumorigenicity tests that can be conducted concurrently.

Toxicology tests can be designed depending on the properties of testing reagents and the purpose of the tests. The Organisation for Economic Cooperation and Development (OECD) Guideline for the Testing of Chemicals [8] is an internationally recognized test guideline for toxicology testing. They should be conducted in a blinded fashion to minimize the bias of measurement and observation by operators. Short-term and long-term end points are to be defined. Toxicology tests should be conducted by using clinically relevant methods of administration so that they can provide insights into a safe range of therapeutic cell doses. Acute (early) and late phase end points should be established in this test.

POC tests often employ a genetically modified animal that offers a model of the disease in question (e.g., Tg, KI, KO or KD mice) or injured animals to address the potential benefit or efficacy of the investigational therapy and to define the range of the effective dose used in clinical application by escalating the doses. The administration route and the method should be as close as possible to the intended clinical use. Positive and negative events should be clearly defined. In such a POC study, indices such as physiological recovery of lost function or overall survival of transplanted cells that could underlie intended therapeutic use are examined. Measurement of indices should be conducted in a blinded fashion to minimize bias during data acquisition. The size of the test group should be large enough to permit meaningful statistical analysis.

Biodistribution tests should be conducted to address tumorigenic proliferation of transplanted cells at the ectopic site. *Alu* sequence PCR is commonly used to detect human cells in host tissues or organs. While this PCR test detects human cells over a 0.1% frequency in host tissue by DNA ratio [9], greater sensitivity is needed to detect small metastatic colonies. In PET technology, proliferative cell mass is labelled by taking in a metabolic probe such as ^18^F FLT, providing a distribution of tumorigenic cell proliferation in the animal’s body. However to trace the behavior of transplanted cells and their biodistribution over time requires labeling test cells by introducing marker genes by retrovirus or lentivirus that can emit a signal with a high S/N ratio. These approaches are currently under development.

## 2. Guidelines for Tumorigenicity Tests

Somatic cells with a normal chromosomal structure show limited proliferation potential. Tumorigenicity testing of mesenchymal stem cells may not reveal a serious problem [10]. However, in the case of PSC-derived cell products, the tumor-forming potential should be examined thoroughly because of the “unlimited” proliferation capacity of PSC and their genetic instability. However, there is no internationally recognized guideline for tumorigenicity testing of cells used for cell therapy. WHO TRS 878, “Recommendation for the evaluation of animal cell cultures as substrates for the manufacture of cell banks” [11,12] provides a guideline for animal cell substrates used for the production of biological medicinal products, but not for cells used for therapeutic transplantation into patients. Recently, FDA/CBER commented on the issues to be considered for cell-based products and associated challenges for preclinical animal study [13]. The report stated that when tumorigenicity testing of ESC-derived cellular products is undertaken, the tumorigenicity tests should be designed considering the nature of cell products to be transplanted and the anatomical location or microenvironment of the host animal. Tumorigenic test results from the administration of cells through nonclinical routes are not considered relevant as they would not assess the behavior of transplanted cells in the intended microenvironment to which the cells would be exposed. The study design should include groups of animals that have received undifferentiated ESCs, serial dilutions of undifferentiated ESCs combined with ESC-derived final products to infer the contamination of undifferentiated ESCs in the final product.

The aforementioned summarizes current discussions of tumorigenicity testing. However, we still need to answer a fundamental question: “How can we extrapolate animal tumorigenicity testing to humans?” The design of tumorigenicity tests should attempt to answer this question. For this, we must first estimate the risk that we will underestimate the incidence of tumor-forming events in humans by conducting an improper or non-informative animal study. So, how do we define such risk? For example, there is a risk that a study is unable to link unexpected tumor formation to genetic abnormalities of test cells presented before transplantation due to inadequate genetic information regarding test cells. In addition, there is a risk of obtaining “false” negative results by transplanting an insufficient dose, using an inadequate monitoring period, using an improper immunodeficient animal model that is insufficient to detect tumor, not transplanting into the right anatomical position, failure of transplantation itself or unexpected early death of transplanted cells in host tissue. We can address the risks by conducting quality control tests of test cells prior to transplantation and small scale pilot studies to determine the design of tumorigenicity tests. The following points should be considered in designing tumorigenicity tests.

1. The history of cell production (cultured in a research lab or Good Manufacturing Practice (GMP) grade cell processing facility).
2. Quality control records of test cells (e.g., phenotype, gene expression, sterility tests, genetic information, passage number and growth rate).
3. The type of immunodeficient animal model used and the route of administration (clinical route or subcutaneous route).
4. The method of transplantation (e.g., embedded with Matrigel or in sheets or in cell suspension).
5. Gender and number of animals to be used.
6. Information about the microenvironment at the transplanting site.
7. Dose of cells to be transplanted.
8. Selection of a positive control cell and definition of positive tumor-forming event.
9. Monitoring periods.
10. Protocol for immunohistochemistry (IHC) to detect transplanted cells in host tissue.
11. Method to detect ectopic tumor formation.

## 3. Specification of Test Cells

Cells used in tumorigenic tests should be generated in a manner as close as possible to that intended for clinical use. In this context, it is preferable that cells used for all preclinical tests should be generated in a GMP-grade cell processing facility for clinical use. This approach would minimize bias originating from differences in cell production quality. Several types of data, including gene expression profiles obtained from gene chips or qRT-PCR to assess stem cell-like markers and differentiation markers, phenotypic analysis by flow cytometry, sterility tests, mycoplasma tests, exome sequencing, chromosomal stability tests with comparative genomic hybridization (CGH) array and karyotyping by multi-color banding (mBAND) or fluorescent *in situ* hybridization (FISH) would be valuable. For iPSC-derived cell products, EB formation assays would provide insights into differentiation potential. The results could be used to select “good” clones that demonstrate no differentiation bias or no differentiation resistance. These quality control tests and cell characterization tests are not a part of tumorigenicity testing *per se*. However, the information on starting material should be linked to the results of tumorigenicity testing to render the test results more informative.

In tumorigenicity testing of PSC-derived cell products, one can anticipate several tumor-forming events that include teratoma formation from residual “differentiation-resistant” PSC with normal karyotype, cancer-like progressive tumor formation from cells with abnormal karyotype or acquired genetic variation during culture and tumors with differentiation bias generated from imperfectly reprogrammed cells. To understand the nature of tumor-forming events, the link with results of these quality control tests is indispensable.

## 4. Selection of an Animal Model

In general, if one were to use “non-immunodeficient” healthy animals or “non-immunodeficient” disease model animals for tumorigenicity testing, one would have to administer a large amount of immunosuppressant for long-term monitoring. However, this approach will not always guarantee satisfactory engraftment of xeno-transplants. Primates can be used for tumorigenicity testing as models representative of humans, but this model is more useful for POC tests, not for tumorigenicity tests. Therefore, immunodeficient healthy rodents are widely used for tumorigenicity testing if human cells (final product) are to be used in the test. Large immunodeficient animals like the SCID pig [14] are also available. However, again, the SCID pig model would be useful to address transplantation efficiency of human cells, such as xeno-bone marrow transplantation of human hematopoietic stem cells as a part of a POC study in large animals. They are not cost-effective large scale statistical studies. To conduct tumorigenicity tests with a sufficient number of immunodeficient animals, a rodent model is a reasonable option for the preparation of test cells. Immunodeficient mice such as nude mice (BALB/cA, JCl-nu/nu), SCID mice (C.B-17/Icr-scid/scid), NOD-SCID mice (NOD/ShiJic-scid) and NOG mice (NOD/ShiJic-scid, IL-2Rγ KO) have been widely used for human cell transplantation studies. Prior to the design of tumorigenicity tests, one needs to evaluate the tumor-generating potential of these immunodeficient mouse strains by transplanting various dose of tumorigenic cell lines subcutaneously.

Another well-known transplantation site in rodents is beneath the testicular capsule space. This transplantation model is mainly used to test for satisfactory engraftment of test cells for POC tests, not for tumorigenicity tests. In our hands, it requires elaborate surgical skills and needs at least 10^4^ iPSCs to generate tumors in NOG mice. In addition, tumor formation in the intraperitoneal space is hard to detect from the appearance of mice, thereby preventing statistical studies for tumor-forming events in a timely manner. In our case, the tumorigenic potential of immunodeficient mice was assessed by transplanting various doses of HeLa cells subcutaneously, following recommended procedure stated in WHO TRS 878 [11,12]. The mice were monitored over 12 months, and the TPD50 (minimum dose that can generate a tumor in 50% of transplanted mice) was calculated by the Trimmed Spearman-Karber method for each strain [9]. HeLa cells were used as a representative line of somatic tumorigenic cells with a genetic abnormality. For transplantation, a collagen-based gel lacking nutrients is sometime used to embed cells and to retain them at the designated transplantation site. Importantly, the gel *per se* does not support growth of the transplanted cells at the site. We have used Matrigel^®^ (BD Biosciences, San Jose, CA, USA) to embed cells and to increase their tumor-forming potential [15]. We obtained the following values for the TPD50 for HeLa cells with Matrigel^®^ via a subcutaneous route: Nude, 10^3.5^ (*n* = 120); SCID, 10^2.5^ (*n* = 24); NOD-SCID, 10^2.17^ (*n* = 24); NOG, 10^1.1^ (*n* = 75). It is notable that during the course of experiments covering 9 months of observation, we also observed spontaneous thymomas with a frequency of some 14% in NOD-SCID mice in agreement with previous reports [16], which makes interpretation of tumorigenicity tests with NOD-SCID mice complicated.

**Figure 1:**
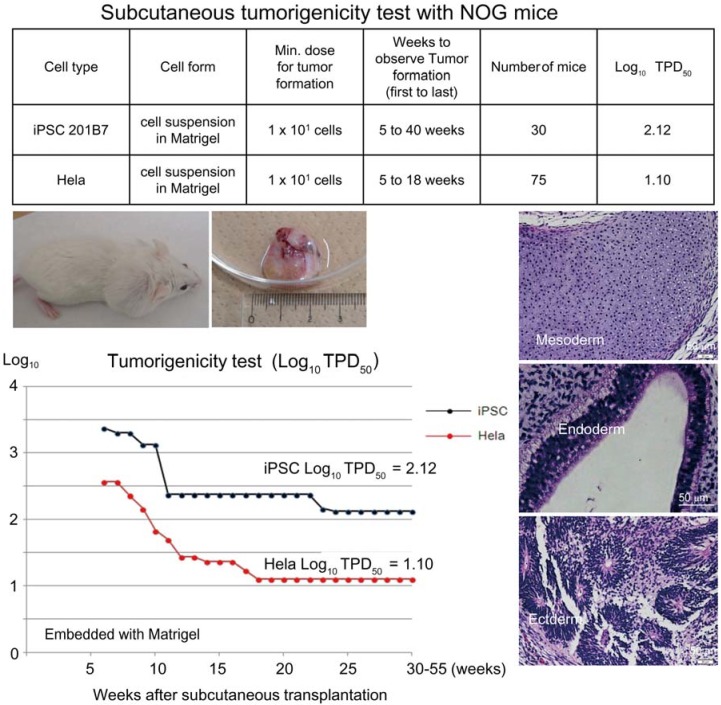
Subcutaneous tumorigenicity test with NOG mice. A table in above showed type of cells used as a positive control for tumorigenicity test (iPSC cell line 201B7 and tumor cell line HeLa), minimum dose for tumor formation and Log_10_ TPD_50_ for them when transplanted subcutaneously with Matrigel^®^. A line graph showed value for Log_10_ TPD_50_ for iPSC or HeLa at respective monitoring point (0–55 weeks). Photos (clock-wise); NOG mouse with tumor, teraoma from NOG mouse, Slice section of teratoma after HE staining; cartilage (mesoderm), intestinal tissue-like (endoderm) or neural rosette-like (ectoderm) tissue.

**Figure 2:**
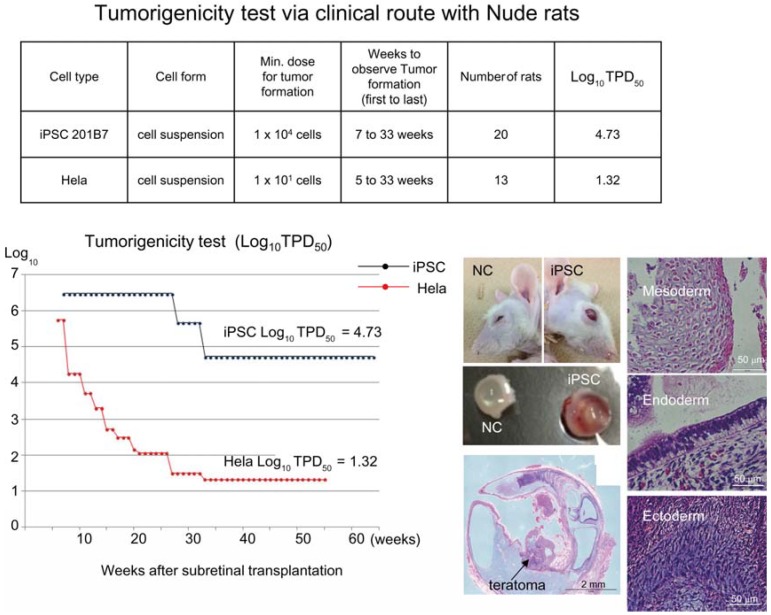
Tumorigenicity test via clinical route with Nude rats. A table in above showed type of cells used as a positive control for tumorigenicity test (iPSC cell line 201B7 and tumor cell line HeLa), minimum dose for tumor formation and Log_10_ TPD_50_ for them when transplanted via clinical route. A line graph showed value for Log_10_ TPD_50_ for iPSC or HeLa at respective monitoring point (0–55 or 64 weeks). Photos (left from top to bottom); NC: non-transplanted control, iPSC: iPSC transplanted mouse. iPSC-transplanted (iPSC) or non-treated control (NC) eye ball. HE staining of slice section of iPSC-transplanted eye ball. Photos (right top to bottom) histology of teratoma formed; cartilage (mesoderm), intestinal tissue-like (endoderm) or neuron-like (ectoderm) tissue.

Based on the preceding data, we chose NOG mice for subcutaneous tumorigenicity testing of iPSC-derived RPE, assuming that NOG mice could generate tumors from the lowest number of residual PSC or tumorigenic non-terminally differentiated PSC-derived cells. We then subcutaneously transplanted various doses of iPSC (201B7, Riken CDB) with Matrigel^®^ into NOG mice to determine TPD50 for iPSC. The TPD50 value for iPSC (201B7) via the subcutaneous route was 10^2.12^ (*n* = 20) over 84 weeks of observation [9] (Figure 1). Tumorigenicity tests via a subcutaneous route with NOG mice is a sensitive quality control test to detect a small number of remaining PSC in PSC-derived investigational product regardless of cell type. Of course, the TPD50 for iPSC transplanted via a clinical route can be checked independently. In our case, we used nude rats for tumorigenicity testing via a clinical route, as the subretinal space of mice is very small and transplanting cells via a clinical route requires outstanding technique by a skilled operator. Thus, we needed larger animals to avoid “false” negative results due to failure of transplantation, to transplant a clinically relevant dose of GMP-grade iPSC-derived RPE (without Matrigel) and to confirm that the transplantation of brown colored RPE was in the right position in the albino eye ball of nude rats [9]. We did not use any “AMD” disease model animals [17,18] because they will not recapitulate all the features of human AMD. In human AMD, the macular region is focally affected and the rest of the retinal area is intact. Treatment of human wet-type AMD with an iPSC-derived RPE sheet is conducted by transplanting the RPE sheet into the affected lesion after removal of choroidal neovascularization. Thus, we assumed that a transplanted RPE sheet would receive a trans-effect from the intact retina. For that reason, we transplanted the RPE sheets into nude rats with intact retinal function rather the recapitulate the microenvironment of the clinical setting. Thus, the choice of animal should be made depending on the degree of immunodeficiency, anatomical demands and planned clinical manipulation. The TPD50 value for iPSC or HeLa cells via the clinical route was 10^4.74^ (*n* = 26) or 10^1.32^ (*n* = 37) respectively (Figure 2). The large discrepancy between the TPD50 values for iPSC and that of HeLa prompted us to examine the effect of the microenvironment on iPSC-derived products to better design tumorigenicity tests via the clinical route (see below).

Another option to address the tumorigenic potential of autologous iPSC-derived products is to transplant rodent cells into a rodent with same genetic background to evade immune rejection associated with xeno-transplantation. Of course, it will be necessary to accumulate sufficient data to demonstrate that rodent cells used in this test are equivalent to human investigational cell products before starting the test.

## 5. Administration Route and Microenvironment at the Transplantation Site

The administration route should mimic the clinical route as closely as possible to address the tumorigenic potential of investigational cells in the context of the microenvironment at the transplantation site. Therefore, evaluation of the microenvironment of the transplantation site including trans-effects from the microenvironment on investigational cells should be assessed prior to the commencement of large scale tumorigenicity testing. In the event of teratoma formation by residual undifferentiated PSCs, trans-effects of host tissue on PSC should be examined. Towards this end, we have established an *in vitro* co-culture system by placing PSC in culture inserts and culturing host or human primary tissue on the bottom of the dish. When iPSCs in culture inserts were co-cultured with cardiomyocytes or neural cells in the bottom of the dish, the growth of iPSC was not affected, but when they were co-cultured with RPE, the number of iPSCs was reduced drastically [19]. We found that RPE secreted Pigment Epithelium-derived Factor (PEDF). Addition of anti-PEDF antibody into the co-culture system blocked the reduction of iPSC cell number. Further addition of recombinant human PEDF (hrPEDF) induced apoptotic cell death and dramatically reduced ESC and iPSC cell number. hrPEDF did not show any reduction in the number of HeLa cells. Indeed, the TPD50 for iPSC was 10^4.75^ when transplanting into the subretinal space (clinical route), while that for HeLa was 10^1.32^. That means that approximately 20 HeLa cells could generate a tumor in the subretinal space in half of the rats transplanted, but more than 5 × 10^4^ iPSCs were required to generate teratomas in the subretinal space in half of the rats transplanted. As we transplanted 0.8–1.5 × 10^4^ iPSC-derived RPE cells in sheets via the clinical route in tumorigenicity tests, it is unlikely that we could observe teratomas from tumorigenicity tests via the clinical route. Further tests, such as transplanting serial dilutions of iPSC in the final product in the subretinal space would not be informative and cannot be justified if tried. However, tumorigenicity tests via the clinical route could be useful to address the tumorigenic potential of non-terminally differentiated tumorigenic cells in iPSC-derived RPE products. This test would be sensitive enough to detect tumors in half the rats transplanted with 20 HeLa cells. We conducted this test for this reason and observed no tumor-forming event (*n* = 36) during a 10–20 months monitoring period. The lack of tumor-forming events was eventually confirmed by IHC of transplanted cells in host tissue section.

We point out that the risk of teratoma formation by a small number of residual iPSC in iPSC-derived RPE in a clinical setting should be thoroughly addressed especially for autologous cell transplantation. Towards this end, subcutaneous tumorigenicity tests are being conducted concurrently with NOG mice wherein we transplant 1 × 10^6^ cells embedded in Matrigel. This test is sensitive enough to detect as few as 10 iPSCs [8]. We have conducted this test with 71 animals that were monitored for 9 to 21 months and obtained negative result after examination of tissue sections by IHC.

In addition, we reported a highly sensitive residual hiPSC detection method based upon qRT-PCR using primers for the *LIN28A* transcript [20] in hiPSC-derived RPE. This method enabled us to detect residual hiPSCs down to 0.002% of differentiated RPE cells. These assays were effective quality control tests and test cells with negative results with this qRT-PCR test could be used for tumorigenicity testing and therapy. We conclude that even if a few (less than 10) autologous iPSCs are present in an iPSC-derived cell product, the chance of developing a teratoma is negligible when transplanted into the subretinal space.

## 6. Monitoring Period

We subcutaneously transplanted various doses of HeLa cells with or without Matrigel^®^ into Nude, SCID, NDO-SCID and NOG mice and into the subretinal space of nude rats. We also subcutaneously transplanted various doses of iPSC with or without Matrigel^®^ into NOG mice or into the subretinal space of NOG mice. As HeLa cells and iPSC can generate tumors in NOG mice with a relatively small number of cells, a long observation period can be required so that a tumorigenic event originating from a small number of transplanted cells is not overlooked. Ten HeLa cells needed 18 weeks and 10 iPSCs needed 40 weeks to generate tumors in NOG mice in the longest cases. Ten HeLa cells needed 33 weeks and 1 × 10^4^ iPSCs required 33 weeks to generate tumors in the subretinal space of nude rats in the most protracted cases. Overall, it is recommended that the immunodeficient rodents be monitored up to 12 months so that a tumor formation event is not missed and to conduct satisfactory statistical analyses.

## 7. Detection of Transplanted Cells

Tumor formation by transplanted human cells can be detected regardless of cell type (teratoma or tumor) by staining tissue sections of the transplant site in host animal with human-specific antibody and Ki67. Nuclear staining with DAPI or Hoechst will not demonstrate that the cells in the tissue section were viable at the time of sacrifice, but sharp margins of the nuclear membrane will suggest that cells were alive and free from autophagy or necrotic events. Human-specific antibodies such as STEM121 (StemCells, AB-121-U-050), Lamin A + C (Abcam, AB108595), and HNA clone 3E1.3 Millipore MAB4383) can be used to identify human cells in host tissue. *In situ* hybridization with a species-specific (human, mouse, rat, *etc.*) probe may generate clear signals, but it may require elaborate sample preparation steps when a paraffin section is used. Tumor-forming cells with proliferation potentials were clearly distinguished by positive staining with Ki67 (MIB-1, Dako M7240) [9]. Further staining of human cells with antibodies specific for human differentiation markers will clearly identify the transplanted human cells.

## 8. Dose, Number and Sex of Immune Deficient Animals

The dose used in tumorigenicity tests should be determined in the context of the intended clinical use. In general, toxicology tests or POC tests require an escalation of doses to define the safety margin or the effective therapeutic margin. However, this may not be the case with tumorigenicity tests as they aim to address the tumorigenic potential of the maximum dose of the cell product that will be used in therapy. Considering the body size of the animal and anatomical space of the receptive transplant site in the animal, a relevant dose should be administered via the clinically route. In our case, we transplanted 0.8–1.5 × 10^4^ iPSC-derived RPE cells into the subretinal space of nude rats and 1 × 10^6^ iPSC-derived RPE cells with Matrigel^®^ subcutaneously, based on the fact that we intended to transplant 4–8 × 10^4^ iPSC-derived RPE in the clinic. We transplanted a maximum or supra-maximum test dose to minimize the risk of underestimating tumor-forming events in a clinical setting.

The number of rodents in each group should be more than 6 for statistical analysis to obtain significant results using the Clopper-Pearson method. If the cell therapy focuses on a single gender, the sex of mice should be matched in the tumorigenicity test. If not, female mice should be chosen to conduct the tests as stated in WHO TRS 878. Male mice attack cage mates, which leads to a reduction of animal number during long-term monitoring.

## 9. Conclusions

It is important to design animal tumorigenicity tests so that they do not underestimate the frequency of tumorigenic events in a clinical setting, based on risk assessment of the respective test. In this review, we have highlighted points to be considered by emphasizing the possible risks and the countermeasures we have taken against them. It is important to gather genetic information from the PSC-derived cell product by CGH array, mBAND and FISH analysis in a timely manner. We need to evaluate the effect of the microenvironment on test cells at the transplant site and the tumor-forming potential of test animals via both the clinical route and via the subcutaneous route. The latter would serve as a sensitive quality control test. This analysis must be mindful of the required dose, type and duration of monitoring and application of an effective IHC method to detect and evaluate the transplanted cells. Conducting pilot studies will help to obtain some of the information and design informative pivotal tests. Clinical researchers need to fully understand the scope and limit of each preclinical test to predict adverse events in the clinic.
